# Entrapment of a Dormia Basket in the Cystic Duct: Case Report

**DOI:** 10.1155/2012/731230

**Published:** 2012-10-11

**Authors:** Dario Pariani, Giorgio Zetti, Ferdinando Cortese

**Affiliations:** Azienda Ospedaliera Ospedale di Circolo di Busto Arsizio, Presidio Ospedaliero di Saronno, U.O. Chirurgia Generale e Toracica, Piazzale Borella, 21047 Saronno, Italy

## Abstract

Nowadays endoscopic treatment of common bile duct stones is considered the treatment of choice for all common bile duct stones. Although this procedure is related to a good success rate, in rare cases serious complications can happen, especially if you use a Dormia basket. Here we describe the clinical case of a patient affected by hepatolithiasis, cholelithiasis, and common bile duct lithiasis with entrapment of a Dormia basket in the cystic duct. It was necessary to perform a surgical choledochotomy to deal with this rare complication.

## 1. Introduction

Since Classen, Demling, and Kawai described the first endoscopic papillotomy in 1974, endoscopic treatment of common bile duct stones has come a long way and is now considered the treatment of choice for all common bile duct stones. After endoscopic papillotomy, 85% to 90% of all common bile duct stones can be extracted using a Dormia basket or balloon catheter [1].

Basket impaction or rupture of the basket traction wire are potential complications. A study collected from expert centers reported that the rate of complications associated with the use of Dormia basket was 3.6% [2]. Here, we report the case of a patient affected by hepatolithiasis, cholelithiasis, and common bile duct lithiasis with a Dormia basket entrapped in the cystic duct.

## 2. Case Report

We present the case of a 47 year-old male patient, arrived in the emergency room for recent onset of epigastric pain associated with nausea, vomiting, dark urine, grey feces, and sclero-skin jaundice.

Blood tests showed serum total bilirubin 8.8 mg/dL, direct bilirubin 6.87 mg/dL, alanine transaminase (ALT) 528 U/L, aspartate transaminase (AST) 175 U/L, *γ*-glutamyltransferase (GGT) 1224 U/L, lactate dehydrogenase (LDH) 360 U/L, and alkaline phosphatase (ALP) 374 U/L.

Abdominal ultrasound showed gallstones in the gallbladder, gallstones and dilation (9 mm) of the common bile duct, and gallstones in the right intrahepatic bile duct.

Magnetic resonance cholangiography (MRC) was performed and confirmed the presence of cholelithiasis and dilation of the common bile duct with an inside prepapillar stone and another one at the carrefour with the cystic duct, dilation (8 mm) of the right intrahepatic biliary tree with branch to segment VI containing numerous stones.

The patient was therefore subjected to endoscopic retrograde cholangiopancreatography (ERCP), during which, after papillosphincterotomy and extraction of the prepapillar stone, in the attempt to catch the proximal stones, after opening the Dormia basket, it was entrapped in the common bile duct near the outlet of the cystic duct.

**Figure 1:**
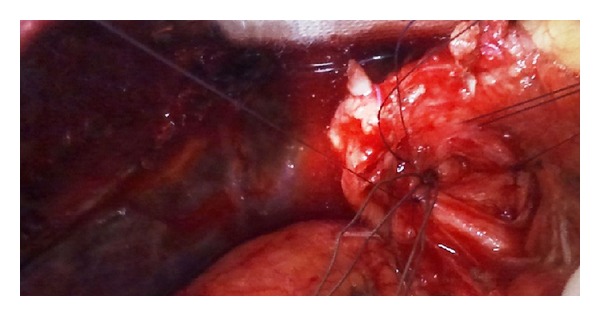
Intraoperative picture showing Dormia basket entrapped in the cystic duct.

After some unsuccessful attempts to extract the basket endoscopically, the patient underwent emergency surgery. Through an under-the-rib trasversal laparotomy, a choledochotomy was performed and it was seen that the banches of the Dormia basket were trapped at the entrance of the cystic duct (Figure 1). Under direct vision the branches were cut and the basket was extracted.  Passing through the choledochotomy, a Fogarty catheter entered the right hepatic duct and managed to extract the intrahepatic stones with no stones left at the intraoperating choledochoscopy.

**Figure 2:**
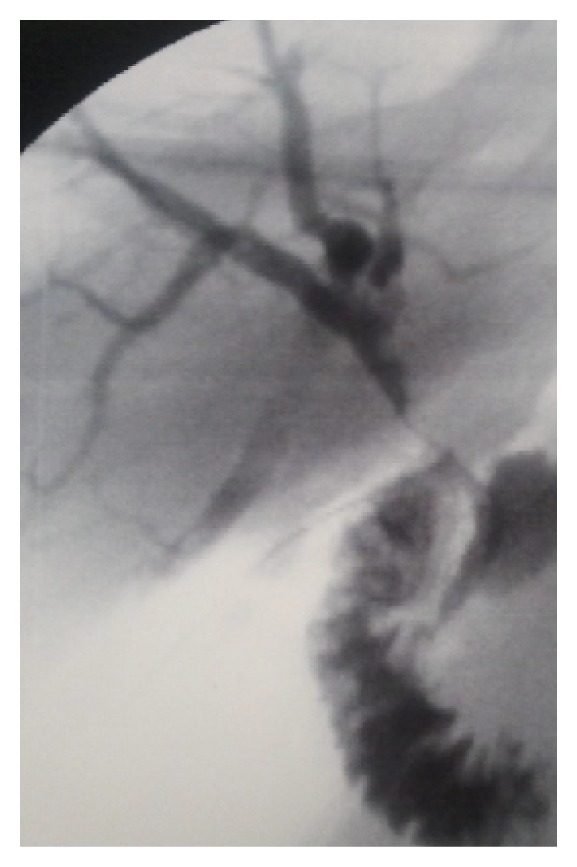
Trans-kher cholangiography at the end of the surgical procedure: no evidence of any filling defects in the biliary tree and good passage of the contrast medium into the duodenum.

At the end of the surgical procedure a cholecystectomy was performed and the choledocus was sutured by interposing a 12 Fr Kher tube with no evidence, at the trans-kher cholangiography, of any filling defects in the biliary tree and showing good passage of the contrast medium into the duodenum (Figure 2).

The postoperative course was uneventful with progressive normalization of the hepatic stasis parameters and of transaminases.

The patient was discharged on the fifth postoperative day in good health conditions and the kher tube was removed on the twenty-first postoperative day after trans-kher cholangiography showing no extraluminal spills of the contrast medium and no traces of gallstones residues in the biliar tree.

## 3. Discussion

Hepatolithiasis, a highly prevalent disease in East Asian countries, is becoming increasingly common in Western populations.

Surgical resection remains the definitive treatment of hepatolithiasis because its goals include the complete removal of intrahepatic stones and the simultaneous resolution of accompanying strictured bile ducts. Surgery may also reduce the risk of recurrent stone formation, cholangitis, and the development of cholangiocarcinoma.

Both ERCP and percutaneous transhepatic cholangiography (PTC) are invaluable tools in the diagnosis and treatment of hepatolithiasis and have the therapeutic ability to allow for the extraction of stones, biopsy of intraductal lesions, and implantation of stents [3].

The treatment of common bile duct stones has advanced from choledochotomy to endoscopic management, with a success rate of over 90% for the latter [4].

The techniques used for the management of common bile duct stones are endoscopic sphincterotomy and endoscopic papillary balloon dilatation. However, the complications of endoscopic management are hemorrhage, pancreatitis, sepsis, cholangitis, and occasionally, impaction of the lithotriptor basket during the endoscopic removal of a stone.

The reported incidence of impaction of a basket with an entrapped stone was 5.9% [5, 6]. When the entrapped stone resists, all four branches of the basket can be stressed, which can result in fracture of the traction wire in up to 5% of cases.

Fracture of the traction cable is a more severe complication of mechanical lithotripsy, which usually requires additional procedures, such as extracorporeal shock wave lithotripsy (ESWL) or surgery. When this occurs with the wire fracture outside the mouth, exchanging the initial 80-cm metal sheath for a shorter ones (70, 60, 50 cm) may allow immediate continuation of lithotripsy in most cases, making this technique time-saving, less expensive, and more successful, avoiding other unnecessary procedures such as ESWL or surgery [7].

In our reported case, the Dormia basket was entrapped in the cystic duct without stones inside of its branches, thus precluding the possibility of using ESWL or other techniques in order to extract the basket easily. Furthermore, in this case, any attempt of extraction of basket would have caused a possible lesion of the common bile duct. Therefore, in rare cases like this, we suggest to proceed with surgery in order to remove, by choledochotomy, the entrapped basket. Furthermore, in this particular case, the surgical approach enabled us to proceed with the removal of intrahepatic lithiasis with good results in the short and medium terms.

## References

[1] Seitz U, Bapaye A, Bohnacker S, Navarrete C, Maydeo A, Soehendra N (1998). Advances in therapeutic endoscopic treatment of common bile duct stones. *World Journal of Surgery*.

[2] Thomas M, Howell DA, Carr-Locke D (2007). Mechanical lithotripsy of pancreatic and biliary stones: complications and available treatment options collected from expert centers. *American Journal of Gastroenterology*.

[3] Sakpal SV, Babel N, Chamberlain RS (2009). Surgical management of hepatolithiasis. *HPB*.

[4] Attila T, May GR, Kortan P (2008). Nonsurgical management of an impacted mechanical lithotriptor with fractured traction wires: endoscopic intracorporeal electrohydraulic shock wave lithotripsy followed by extra-endoscopic mechanical lithotripsy. *Canadian Journal of Gastroenterology*.

[5] Schneider MU, Matek W, Bauer R, Domschke W (1988). Mechanical lithotripsy of bile duct stones in 209 patients—effect of technical advances. *Endoscopy*.

[6] Sauter G, Sackmann M, Holl J, Pauletzki J, Sauerbruch T, Paumgartner G (1995). Dormia baskets impacted in the bile duct: release by extracorporeal shock-wave lithotripsy. *Endoscopy*.

[7] Chan SS (2010). How should biliary stones be managed?. *Gut and Liver*.

